# Valproic acid is associated with cognitive decline in HIV-infected individuals: a clinical observational study

**DOI:** 10.1186/1471-2377-6-42

**Published:** 2006-12-06

**Authors:** Lucette A Cysique, Paul Maruff, Bruce J Brew

**Affiliations:** 1 Department of Psychiatry, University of California at San Diego, San Diego, California, USA; 2 Centre for Neuroscience, University of Melbourne, Australia; 3 Departments of Neurology and HIV Medicine, St Vincent's Hospital, Sydney, Australia

## Abstract

**Background:**

Valproic acid (VPA) is often used to control pain in HIV-related neuropathy. However, the effect of VPA on cognitive functions in advanced HIV-infected individuals is largely unknown. A recent study would suggest that it may have a neuroprotective effect, the doses used were low and the observation period short.

**Methods:**

We used a well studied HIV-infected cohort assessed for a median of 15 (range 6–27 months) to determine whether individuals who were receiving VPA showed any cognitive benefits. Multiple regression procedures allowed us to control for the effects of HAART and HIV disease status as well as numbers of visits and variation in VPA intake over-time.

**Results:**

We found a negative effect of VPA (mean dose of 850 mg/d for 18 months on average; range 6–27 months) on cognitive performance in eight advanced HIV-infected individuals compared to 32 advanced HIV-infected individuals on no VPA who had comparable neuropsychological performance at baseline. Control for plasma HIV viral load provided similar results.

**Conclusion:**

Our results suggest that further studies of VPA in advanced HIV-infection should cautiously include high doses over prolonged periods of at least 18 months in order to more accurately determine whether the posited neuroprotective benefit of VPA still occurs or whether it is replaced by toxicity.

## Background

There is now some evidence that despite the introduction of highly active antiretroviral therapy (HAART), HIV-associated neurocognitive disorders remains common [1]. Moreover, with longer survival, HIV-infected individuals require complex management of co-morbid neurological diseases. Sensory neuropathy is the most frequent neurological complication of advanced HIV-infection and Valproic acid (VPA) is often used to control the associated pain [2]. In a small proportion of HIV-infected individuals VPA is prescribed to control HIV-related epilepsy [2]. The effect of VPA on neurocognitive functions in advanced HIV-infected individuals is largely unknown.

In a healthy population, it has been shown that VPA causes only transient and very mild or no cognitive deficits [3,4]. But, these results are derived from short-term studies and the long-term effects have not been thoroughly investigated. Moreover, it has been suggested that VPA may be associated with more cognitive slowing due to its "sedating" effects related to a predominance of potentiation of gamma-aminobutyric acid (GABA) inhibitory neurotransmission [5]. This may be especially relevant to patients with HIV-associated neurocognitive impairment where cognitive slowing is one of the cardinal features.

However, a recent clinical study suggests that there may be a neuroprotective effect of VPA as an adjunctive therapy to antiretroviral treatment. Using a 10-week placebo controlled study, the authors found a non-significant trend for better cognitive functioning in the VPA group (250 mg twice a day) on cognitive function in advanced HIV-infected individuals [6]. This is consistent with results of in vitro studies showing a neuroprotective effect of VPA in a murine model of HIV-1 encephalitis [7]. While these results are potentially important, several methodological issues render their interpretation difficult. The effect of VPA was only observed over the short-term in a small group, while HAART duration and number of neuroactive drugs in the HAART regimen were not documented. In addition, the effect of HAART was not investigated in the analyses [8].

We have been investigating the effects of HAART on cognitive function in a well studied HIV-infected cohort studied over a long period of time [9]. Given the potential importance of the VPA finding in the Schifitto et al. study [6], we used our observational study cohort to determine whether individuals who were receiving VPA showed any cognitive benefits, while accounting for the effects of HAART and HIV disease status.

## Methods

### Subjects

The cohort of advanced HIV-infected individuals, participating in a longitudinal study of the neurocognitive effect of HIV infection, was previously described in detail in Cysique et al. [1,9] Among 101 HIV-infected individuals with stage C3 HIV disease, 81 came for a second visit at six months and at that time, seven HIV-infected individuals were treated with VPA (average of 850 mg/d for 18 months; range 6–27 months) for HIV-related painful neuropathy and one for HIV-related epilepsy. Patients had been on VPA for at least three months before enrolment. At the time of assessment, all had neuropathy and epilepsy symptoms under control as assessed by an experienced neurologist (BJB). Five of these individuals came for a third visit at 15 months after baseline and four for a fourth visit at 27 months after baseline. All were on VPA at session 2 (8/8); 3 at session 3 (3/5) and, 2 at session 4 (2/4). Continuation or discontinuation of treatment was related to the clinical assessment of neuropathy symptoms. The individual with HIV epilepsy came back at one follow-up only (see also Table 1).

In order to adjust for baseline neuropsychological performance between the individuals on and off VPA, we selected a sub-group of the individuals who were not receiving VPA and who had comparable neuropsychological performance. To accomplish this, we computed a summary z-score corrected for age and education (summary of 14 individuals neuropsychological tests, see [1,9] for additional information) and defined the inter-quartile performance range in the group on VPA (i.e., z-summary score = -0.49 to -1.58). Forty individuals off VPA performed at baseline within this defined range and were used in the following analyses. Consequently, there was no difference in baseline performance between the individuals on and off VPA (*p *= .58).

At baseline, the eight subjects receiving VPA did not differ from the 32 individuals off VPA in terms of age (48.3 years old ± 13 versus 51.9 ± 9 years old, respectively), education (13.8 years ± 5 versus 13.6 years ± 3, respectively), HIV duration (10.8 years ± 5.4 versus 11.5 years ± 5, respectively), nadir (47.5 cpy/mL ± 48 versus 90.2 cpy/mL ± 60.1, *p *= .06, respectively) and current CD4 (367.6 cpy/mL ± 265.6 versus 324.5 cpy/mL ± 240.4, respectively, log _10 _plasma viral load (2.3 ± 1.2 versus 3.2 ± 1.5, *p *= .12, respectively), and year of 1^st ^HAART (1996.5 in both groups) and number of AIDS-defining illnesses (25% had more than two AIDS-defining illnesses in the on VPA group, while 16% had had more than two in the non VPA group, *p *= .53). Across the study period the proportion of participants for whom their HAART regimen was updated did not differ between the groups on and off VPA. However, the eight subjects had proportionally more past HIV-related CNS disease compared to the patients who were not on VPA at session 2 (62.5% [5/8] versus 9.4% [3/32]; *p *< .01). These were five cases of mild HIV-associated Dementia and one case of Cryptococcal Meningitis; all had clinically resolved at least six months before study entry (This was a criterion of exclusion/inclusion in the original study, see [1,9] for details). Furthermore, at baseline the individuals on VPA did not report more depressive complaints than the individuals who were not on VPA (*p *= .18).

### Procedure

All individuals were assessed with a standard neuropsychological test battery encompassing ten tests and eight cognitive domains (learning, memory, processing speed, attention, complex attention, motor functions, language and, visuo-spatial abilities (See [1,9] for additional details).

Our research conformed to the Helsinki Declaration outlining the principles for medical research involving human subjects. All subjects completed an informed consent form prior to study participation. In addition, the University of New South Wales and the St. Vincent's Hospital (Sydney, Australia) research committees approved the research protocol.

### Statistical Analysis

Because we were primarily interested in the cognitive change over-time, the focus of our previous study [9], we determined a composite reliable index of change (RCI). The RCI method has been shown to be equivalent to more complex regression models [10] in determining change in cognitive function. Here we use a newly developed RCI described in detail in Mollica, Maruff & Vance [11] and [9]. The composite RCI was based on six neuropsychological measures that were found to be the most sensitive to HIV-related cognitive decline (See [9] for additional details). Change in cognitive functions was assessed from the individuals who returned for session 2 (32 not on VPA and 8 on VPA). The individuals on VPA and not on VPA were directly compared on demographic, laboratory and clinical variables at baseline. Between-groups analyses were conducted with Fisher's Exact Test or t-test when appropriate.

Moreover, to account for the variability in the number of visits and VPA intake across time, we used a multiple regression (or Analysis of Covariance – ANCOVA) procedure described by Bland [12]. This allows controlling for these effects on the cognitive performance over-time. One factor that varied over-time (depressive complaints) showed a difference (*p*=.05) between groups at session 2 and was therefore included as covariate in the regression model. Lastly to account for change in the HIV disease status as well as HAART update and efficacy, we conducted an additional ANCOVA with log plasma viral load as a covariate.

## Results

**Figure 1:**
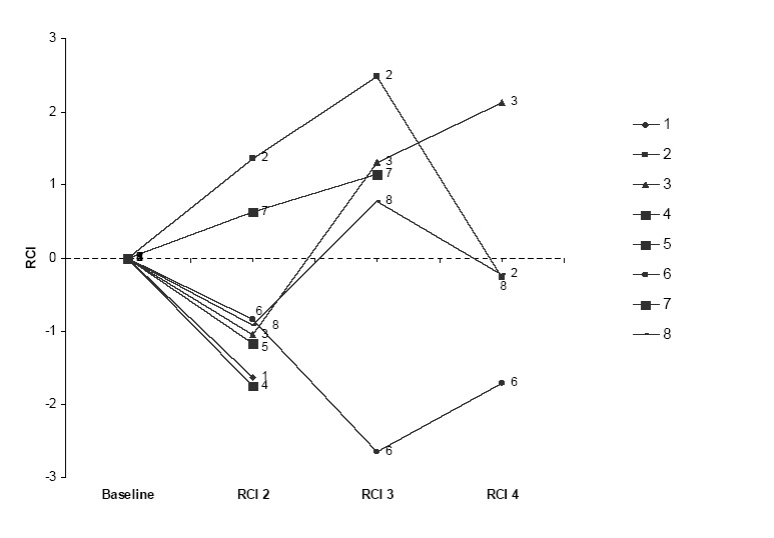
Neuropsychological performance (composite RCI) in the 8 cases on VPA at the second follow-up visit. All cases were on VPA at session 2, cases 2 and 8 were off VPA at session 3 and case 3 and 8 were off at session 4. A negative composite RCI indicates decline.

Over time, we found greater cognitive impairment in individuals on VPA (r = -0.42; *p *< .005). With the effect of depressive symptomatology on cognitive performance controlled, VPA-related cognitive impairment remained statistically significant, albeit reduced (r = -0.34; *p *< .03). Figure 1 illustrates the neuropsychological performance over-time in the eight cases. When HIV disease status (plasma viral load was used as a surrogate marker of HIV disease and HAART efficacy) was added to the statistical model the negative relationship between VPA and cognitive function remained unchanged (i.e., r = -0.44; *p *< .005).

## Discussion

Over time, we found greater decline in cognitive function in individuals who were receiving VPA at the time of their follow-up examination (at six months, 15 months and 27 months after baseline). This decline could not be explained by the presence of depressive complaints or by HIV disease severity; defined by plasma viral load. Furthermore, the eight patients in our cohort who were receiving VPA were more likely to have been diagnosed with a past HIV-related CNS diseases compared to individuals not receiving VPA. Our results support a relationship between cognitive decline and VPA, although, further study is required to determine the extent and magnitude of this relationship. For example, if our results are replicated then it is possible that the diminished cognitive reserve arising from advanced HIV CNS pathology renders cognitive function more susceptible to the deleterious effect of VPA.

Importantly, in addition to comparable baseline neuropsychological performance, our groups were well-matched on demographics as well as HIV-related disease variables reinforcing the robustness of a negative effect of VPA on cognitive functions in advanced HIV-infected individuals.

Contrary to previous studies in healthy [3,4] and HIV-infected individuals [6], our study assessed the effect of VPA on cognitive functions with higher dose of VPA and over a longer period of time in individuals whose disease status put them at increased risk for cognitive decline. It is possible that due to non-randomization and no control of the group assignment, the effect observed was an artifact of advanced HIV-infection. While, this possibility remains to be fully investigated, the complete results of our longitudinal study are not supportive of such an interpretation as most individuals with advanced HIV-infection had stable cognitive performance over the time period studied here (see [9]). Our study included a small number of subjects who also decreased over time, reducing the generalizability of our results. The Schifitto et al. study [6] has also included a similarly small number of individuals, while none of their results was statistically significant. Nevertheless, we chose to explore the VPA effect with hierarchical regression. This statistical technique allowed us to use cases with different numbers of re-assessments and to control the effects of disease severity and depression as well as correct for the drop-out over-time even in our small sample. It also allowed us to accommodate variation in VPA intake over-time which is common feature of its use as a therapy for co-morbid CNS disorders in advanced HIV-infection.

Despite these limitations, the results highlight three important points that have not been mentioned in any related studies thus far and which should be factored into subsequent trial designs. VPA may be detrimental:1) in doses at the higher end of the therapeutic range, 2) over prolonged periods, and 3) in patients with relatively advanced HIV disease.

There are a number of potential explanations for the association between VPA and cognitive deterioration in advanced HIV-infection observed here. It was originally proposed that VPA had an adverse effect on HIV disease by increasing HIV viral load but our results are not supportive of this explanation [13]. Another possibility is that VPA is having an adverse effect on mitochondria [14]. This is certainly known to be the case in children. Carnitine supplementation is recommended if VPA is used in patients with mitochondrial disorders [15]. It is possible therefore that VPA is detrimental to cognition through a "triple hit" to mitochondria: HIV is known to affect mitochondrial function, some antiretrovirals also compromise mitochondrial function, and VPA is known to impair mitochondrial function. While these alone or in limited combination may not be enough to disturb mitochondrial function the presence of all three may act additively especially in those patients with the potential for limited cognitive reserve.

## Conclusion

Altogether, these results demonstrate that the hypothesized neuroprotective effect of VPA mainly derived from in vitro studies [7,16,17] should be considered with additional caution until larger studies are conducted. In future clinical studies the sample size should be larger and longer term with perhaps surrogate measures of brain mitochondrial function (magnetic resonance spectroscopy – MRS) included. The use and duration of the baseline HAART regimen as well as probably the number of brain penetrating antiretroviral drugs should be balanced between treatment groups in order to control for a potential beneficial effect of HAART.

## Competing interests

The author(s) declare that they have no competing interests.

## Authors' contributions

Initial conception of the study was undertaken by BJB. LC: collected the data. All authors participated in the design of the study. LC conducted the statistical analyses under the supervision of PM. LC, PM & BJB: drafted the manuscript. All authors read and approved the final manuscript.

## Pre-publication history

The pre-publication history for this paper can be accessed here:



## Figures and Tables

**Table 1:** demographic, clinical and laboratory characteristics of the eight HIV+ individuals at baseline and session 2

**Baseline**									
**Patients**	**VPA**	**HIV duration**	**Nadir CD4**	**Current CD4**	**Log Plasma VL**	**VL 50 cp/mL**	**Year of 1st HAART**	**Past HIV-CNS**	**Depressive complaints**

1.00	**1**	11.00	90.00	580.00	1.69	undetectable	1996.00	1	mild
2.00	**0**	16.00	14.00	187.00	2.70	detectable	1996.00	1	mild
3.00	**0**	5.00	144.00	220.00	1.69	undetectable	1996.00	0	moderate
4.00	**1**	11.00	42.00	858.00	1.69	undetectable	1998.00	1	none
5.00	**1**	16.00	10.00	16.00	5.28	detectable	1996.00	1	moderate
6.00	**1**	9.00	25.00	220.00	1.69	undetectable	1997.00	0	mild
7.00	**1**	2.00	50.00	450.00	1.70	undetectable	2000.00	0	moderate
8.00	**1**	17.00	5.00	410.00	2.23	detectable	2000.00	1	none

**Session 2**									

**Patients**	**VPA**	**Current CD4**	**Log Plasma VL**	**VL 50 cp/mL**	**Depressive complaints**	**VPA session 3**	**VPA session 4**		

1.00	**1**	683.00	1.70	undetectable	moderate	**-**	**-**		
2.00	**1**	204.00	2.60	detectable	none	**0**	**1**		
3.00	**1**	240.00	1.69	undetectable	moderate	**1**	**0**		
4.00	**1**	864.00	2.60	detectable	none	**-**	**-**		
5.00	**1**	90.00	4.91	detectable	moderate	**-**	**-**		
6.00	**1**	252.00	2.60	detectable	severe	**1**	**1**		
7.00	**1**	460.00	1.69	undetectable	mild	**1**			
8.00	**1**	260.00	1.69	undetectable	severe	**0**	**0**		
